# Vitamin D intake in Italian healthy subjects and patients with different pathological disorders

**DOI:** 10.3389/fnut.2025.1672798

**Published:** 2025-10-20

**Authors:** Ranuccio Nuti, Luigi Gennari, Guido Cavati, Carla Caffarelli, Bruno Frediani, Stefano Gonnelli, Concetta Laurentaci, Giulia Letizia Mauro, Nazzarena Malavolta, Giovanni Minisola, Maria Punzo, Anna Capozzi, Monica Pinto, Fabio Vescini, Edoardo Conticini, Giammarco De Mattia, Agostino Gaudio, Colin Gerard Egan, Daniela Merlotti

**Affiliations:** 1 Department of Medicine, Surgery and Neurosciences, University of Siena, Siena, Italy; 2 Azienda Sanitaria Matera, Matera, Italy; 3 Dipartimento delle Discipline Chirurgiche, Oncologiche e Stomatologiche, University of Palermo, Palermo, Italy; 4 Casa di Cura Madre Fortunata Toniolo, Bologna, Italy; 5 Ospedale San Camillo, Rome, Italy; 6 ASP Cosenza, Cosenza, Italy; 7 Policlinico Gemelli, Rome, Italy; 8 Istituto Nazionale Tumori IRCCS Fondazione G. Pascale, Napoli, Italy; 9 Department of Oncology, University of Udine, Udine, Italy; 10 Department of Medical and Surgical Specialties, University of Pisa, Pisa, Italy; 11 Department of Clinical and Experimental Medicine, University of Catania, Catania, Italy; 12 CE Medical Writing SRLS, Pisa, Italy; 13 Department of Medical Sciences, Azienda Ospedaliera Universitaria Senese, Siena, Italy

**Keywords:** vitamin D intake, hypovitaminosis D, dietary assessment, chronic disease, Italian population

## Abstract

**Background:**

Vitamin D deficiency is recognized as a widespread public health issue, particularly among individuals with limited sun exposure or chronic diseases. While cutaneous synthesis provides most vitamin D, dietary sources remain essential, particularly in populations with restricted outdoor activity or poor dietary habits.

**Methods:**

This cross-sectional study evaluated dietary vitamin D intake in 1,372 Italian adults (997 females, 375 males; aged 40–80 years) using a validated 14-day Food Frequency Questionnaire (FFQ). Participants included 429 (31.3%) healthy individuals and 943 (68.7%) patients with various pathological conditions. The study was conducted across different Italian regions between May 2023 and December 2024. Analysis of variance (ANOVA) was used to compare differences in vitamin D intake by age, sex, health status, education, occupation, and dietary patterns. Multivariate linear and logistic regression analyses were applied to identify independent predictors of daily vitamin D intake.

**Results:**

Vitamin D intake was low across the entire cohort, with daily means of 198.5 IU (females) and 246.7 IU (males), significantly below recommended levels. Intake decreased with age and was lower in patients than in healthy subjects. The lowest intakes were observed in patients with osteoporosis, renal, oncologic, and neurological conditions. Socioeconomic status and education level were significantly associated with intake levels. Vegetarians and vegans showed particularly low intake levels (152.1 and 83.6 IU/day, respectively). Multivariate regression revealed that male sex predicted higher intake (+44.1 IU/day), while Northern Italian residence (−53.0 IU/day), lower education (−39.2 IU/day), and vegetarian/vegan diets were independently associated with reduced intake. Logistic regression showed male sex was protective against very low intake (< 200 IU/day) (Odds ratio: OR 0.72), while Northern residence (OR 1.61), low education (OR 1.45), vegetarian (OR 1.86), and vegan diets (OR 3.89) increased risk. Age and chronic disease status were not significant independent predictors after adjustment.

**Conclusion:**

This study confirms extremely low vitamin D intake in Italian adults, especially in older adults and those with chronic conditions. Public health initiatives promoting vitamin D-rich diets, food fortification, and supplementation, especially for at-risk groups, are urgently needed to prevent hypovitaminosis D and its associated health consequences.

## 1 Introduction

Hypovitaminosis D is widely recognized as a pathological condition and represents a significant public health problem, involving pregnant women, obese individuals, and subjects who, for various reasons, cannot regularly expose themselves to sunlight (1–3). Indeed, cutaneous synthesis accounts for at least 80% of the requirement, while the remaining 20% is obtained through dietary intake (4–6).

Many factors may contribute to induce a reduction in vitamin D status including physiological factors such as dark skin pigmentation, pregnancy, and age; pathological conditions such as obesity, malabsorptive syndromes, and hepatic/renal failure; medication; and low ambient ultra violet radiation level due to geographic regions of high latitude (7). In the United States pediatric population, where cereals and milk are fortified with cholecalciferol, the presence of vitamin D deficiency is attributed to reduced milk intake, albeit the use of protective creams during sun exposure and the increasing prevalence of obesity may also concur (8).

Moreover, a low vitamin D intake related to low vitamin D diet without fortified food seems to play a significant role. Both D2 (ergocalciferol) and D3 (cholecalciferol) are found in limited amounts in foods: the main sources include mushrooms (exposed to ultraviolet light) and fish, which have the highest natural amount of cholecalciferol (9–11). Beef liver, chicken, pork, egg yolk, cheese, and milk contain reduced amounts of vitamin D, primarily as cholecalciferol and its metabolite 25(OH)D3; foods like fruits, vegetables, rice, and pasta do not contain vitamin D (12).

Recently, we have reported that in the Italian population, the amount of vitamin D intake from foods is limited, with a mean daily intake in females and males of 5.1 μg (202 IU) and 6.2 μg (250 IU), respectively (13). These data are far from the recommended values of 15 μg (600 IU) and 20 μg (800 IU) for subjects aged 51–70 years or above 70 years (7, 9). Vitamin D intake was markedly reduced in subjects following vegetarian or vegan eating habits.

The presence of vitamin D receptors in most tissues and cells, and the discovery that several of them has the possibility to convert 25(OH)D into the active form 1,25(OH)2D, has yielded new insights as regards to the function of vitamin D (14). Beyond calcium, phosphorus and bone metabolism, non-skeletal actions of vitamin D include muscle strength: clinical consequences of hypovitaminosis D are represented by osteomalacia, increased risks of secondary hyperparathyroidism with fragility fractures, muscle weakness with enhanced risk of falls, particularly in elderly subjects (14–16). Moreover, other potential extra-skeletal actions (17, 18) suggest a role for vitamin D in cancer, cardiovascular health, obesity, immunity diabetes, and metabolic syndrome (15, 19–23).

Observational studies have shown lower rates of death from cancer and cardiovascular disease (CVD) in regions with greater sun exposure than in areas with less sun exposure. A significant inverse relationship between baseline serum 25(OH)D and total CVD events as well as CVD mortality has been observed (24–27): a 7% lower risk of total CVD events per 10 ng/mL 25(OH)D increment was reported (28). Confirming this observation, an inverse correlation between 1,25(OH)D and 25(OH)D and risk of CVD was also observed in elderly individuals in Italy (29). In cancer, several epidemiologic studies suggest that circulating calcifediol concentrations may be inversely related to total cancer incidence; moreover, an inverse association of circulating 25(OH)D serum concentration with cancer mortality (30). A relationship was also reported between 25(OH)D levels and pulmonary functions, suggesting that vitamin D deficiency may increase risk of respiratory infections (31).

Hypovitaminosis D is frequent in Italy, as recently seen in cohort studies in the general population as well as in patients with metabolic bone disorders (32). Furthermore, in Italy, the prevalence of low vitamin D is high in elderly women (aged 60–80 years) with values of 25(OH) vitamin D < 5 ng/ml seen in 27% and < 12 ng/ml in 76%, respectively (33).

Our previous analysis of dietary intake patterns in Italian adults confirmed that vitamin D intake is consistently below recommended levels and that inadequate consumption is especially evident among women and older age groups (13). However, to date no study has comprehensively evaluated dietary vitamin D intake across the Italian population while simultaneously considering geographic, socioeconomic, and clinical characteristics.

Therefore, to address this gap, the aim of the present study was to quantify average dietary vitamin D intake in a large sample of Italian adults, identify main food sources, and examine differences by sex, age, geographic area, socioeconomic indicators, dietary pattern, and disease status. Based on our previous observations (13), we hypothesized that (i) mean intake would be substantially below recommended levels; and (ii) intake would be lower among women, older adults, vegetarians/vegans, and participants reporting chronic diseases.

## 2 Materials and methods

### 2.1 Subjects and study design

This was a real-life cross-sectional observational study including 1,372 subjects, comprising 997 females (72.7%) and 375 males (27.3%), aged between 40 and 80 years. Of these, 429 individuals (31.3%) were apparently in good health with no ongoing pathologies, while 943 (68.7%) were affected by various disorders. The only exclusion criteria were ages < 40 and > 80 years. Data were collected using a validated 14-day Food Frequency Questionnaire (FFQ) form: previously, questionnaires collected in 50 healthy adults were compared with results derived in the same population from a designed 14-day frequency food diary (FFD) and we observed that data from FFQ and FFD significantly correlated with each other and were further validated by Bland-Altman plot (34). FFQ reported personal data of subjects such as age (subdivided in decades from 40 to 80 years), sex, occupation, educational degree, region of origin, specific eating habits like vegetarian or vegan, and the presence of osteoporosis, cardiovascular, respiratory, kidney, gastrointestinal, endocrine, neurological, and neoplastic diseases. The survey targeted community-dwelling individuals and was conducted through Clinical Centers affiliated with GISMO (Italian Group for the Study of Bone Diseases) and GIBIS (Italian Group for the Study of Bisphosphonates), located across Northern, Central, and Southern Italy. All participants were fully informed about the study’s objectives and provided written informed consent. The study protocol received ethical approval from the Regional Ethics Committee (Regione Toscana, Sezione Area Vasta Sud Est) and was carried out between May 2023 and December 2024.

### 2.2 Frequency food questionnaire

Food Frequency Questionnaire contained 11 different questions regarding the type and quantity of foods containing vitamin D consumed in the 14 days preceding the interview. Foods were included in FFQ were: milk, corn flakes with vitamin D, yogurt, cheese, meat, fish, egg, food with egg, cured meat, desserts containing egg, milk, or yogurt, and mushrooms. The food intake frequency was evaluated based on how many times that food was eaten in 14 days (from 1 to 14 times). By correlating the amount of each food consumed over the 14-day period with its vitamin D content per 100 mg, it was possible to calculate the quantity of vitamin D in IU (International Unit) ingested through food in 2 weeks. The USDA National Nutrient Database (35) and the CREA database (36) (“Consiglio per la Ricerca in agricoltura e l’analisi dell’Economia Agraria”) were utilized to calculate the vitamin D amount for each food. Data were collected and analyzed using Microsoft Excel. Further details regarding questions on the type and quantity of foods containing vitamin D are specifically reported in our previous paper (13).

### 2.3 Statistical analysis

Data were summarized as means ± standard deviation (SD) or number and%. Probability density functions were also estimated to evaluate the distribution of global and daily vitamin D intake, subdivided by gender and decade of age. Analysis of variance (ANOVA) was used to evaluate the variation in vitamin D intake and different variables including the personal data of the subjects (e.g., age decade, sex, and possible specific eating habits such as vegetarian or vegan) and the presence or absence of pathological conditions.

In addition, multivariable linear regression models were fitted to identify independent predictors of daily vitamin D intake, adjusting for sex, age, geographic region, education level, dietary habits, and chronic disease status. Results are presented as regression coefficients (β) with 95% confidence intervals (CIs) and corresponding *p*-values. To assess factors associated with very low vitamin D intake (< 200 IU/day), multivariable logistic regression was applied using the same covariates. Results are expressed as odds ratios (ORs) with 95% CIs and *p*-values. Robust standard errors were used to account for potential heteroskedasticity.

Assuming a margin of error of 5% and a confidence level of 95%, the recruited sample of 1,372 subjects was considered statistically adequate for the purpose of the study. A *p*-value of *p* ≤ 0.05 was considered statistically significant. All statistical analyses were performed using Statistica 10 (Statsoft, Tulsa, OK, United States) and SPSS (SPSS, version 21.0, IBM Corp., Armonk, NY, United States).

## 3 Results

### 3.1 Population characteristics

A total of 1,372 subjects (997 females, 375 males) from 17 Italian regions participated in the survey (Table 1). The most represented areas included Campania (17.4%), Sicily (15.7%), Lazio (13.3%), Tuscany (10.2%), Calabria (12.0%), and Basilicata (9.8%). Age distribution was balanced across decades from 40 to 80 years, with the 61–70 age group being the most represented (34.3%). Regarding education, 73.4% of participants held either a high school diploma or university degree (36.7% each), while 8.9% and 17.8% had only primary or middle school education, respectively. Higher education levels were more common among younger participants. Employment status showed 30.3% were retired, followed by employees (24.6%), freelancers (12.2%), housewives (13.0%), workers (2.6%), farmers (2.0%), and artisans (1.1%). Overall, 31.3% (*N* = 429) were apparently healthy, while 68.7% (*N* = 943) had one or more chronic diseases.

**TABLE 1:** Features of population included in the study, divided for both males and females into age decades, and educational degree.

Total population			Education							
Age range (years)	Females *N* (%)	Males *N* (%)	Females (*N*)				Males (*N*)			
			P	M	H	U	P	M	H	U
40–50	131 (13.1)	61 (16.3)	–	11	30	90		4	19	38
51–60	281 (28.2)	86 (22.9)	9	47	123	102	1	13	34	38
61–70	351 (35.2)	120 (32.0)	35	72	143	101	4	19	43	54
71–80	234 (23.5)	108 (28.8)	67	60	66	41	6	18	45	39

P, elementary/primary school diploma (8.9%); M, secondary/middle school diploma (17.8%); H, high school diploma (36.7%); U, university degree (36.7%).

### 3.2 Vitamin D intake by sex, region, and age

**FIGURE 1:**
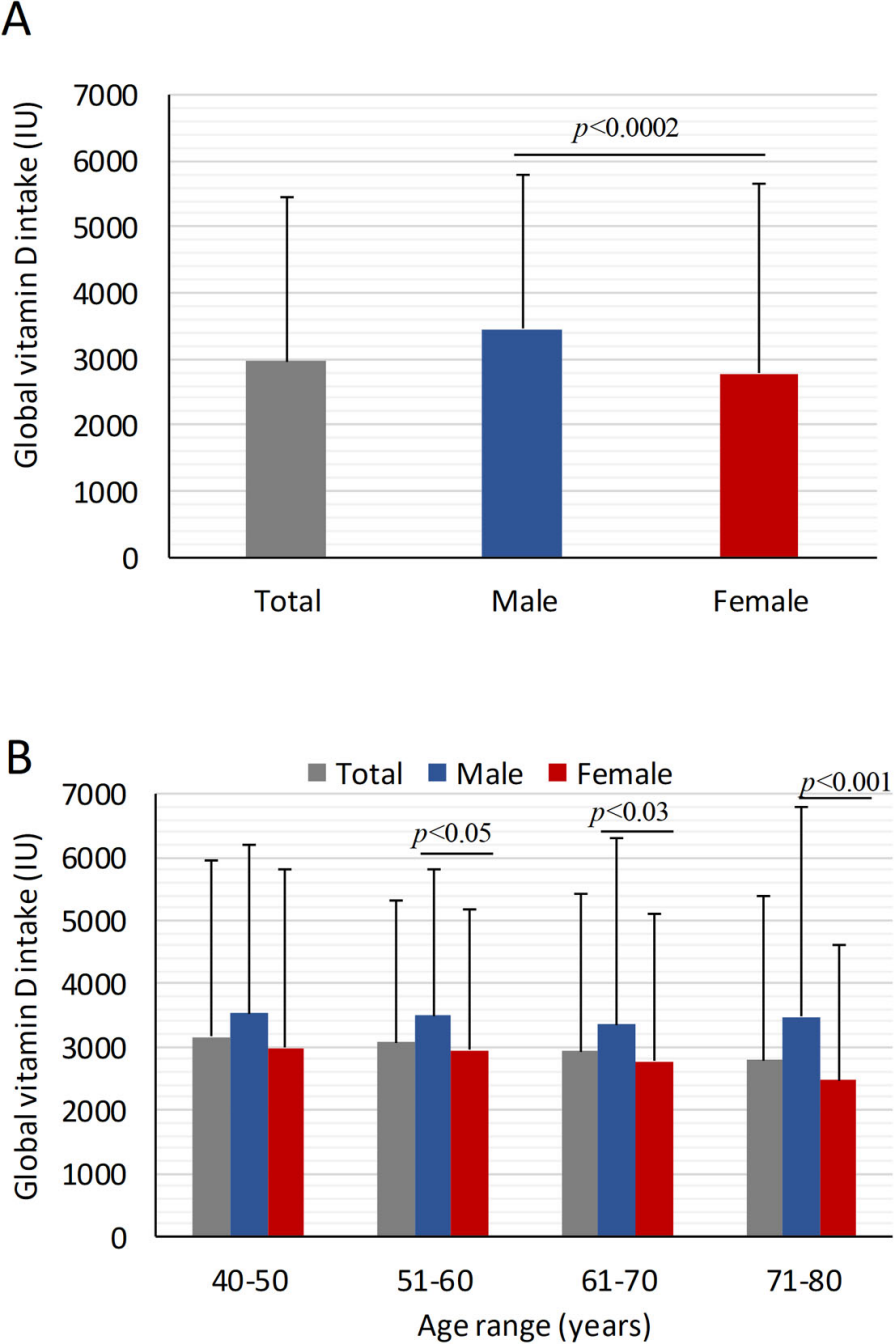
Mean global vitamin D intake in healthy subjects and patients with pathological disorders stratified by gender and age. Global mean vitamin D intake across age decades, stratified for males and females. **(A)** Mean global vitamin D intake over 14 days in all individuals, male and females; **(B)** Global mean vitamin D intake in over 14 days stratified by age decades and gender. The data reveal a progressive decline in vitamin D intake with age, particularly among females. Statistically significant differences between male and female groups for specific age ranges are indicated by *p*-values (*p* < 0.05, *p* < 0.03, *p* < 0.001, *p* < 0.0002). Data are presented as mean ± SD.

The average 14-day vitamin D intake was significantly higher in males (3453.5 ± 2870.8 IU; 95% CI: 3163.0–3744.1 IU) than in females (2779.9 ± 2325.4 IU, 95% CI: 2635.6–2924.3 IU; *p* < 0.002). Daily intake averaged 246.7 ± 205 IU in men and 198.5 ± 166.1 IU in women, both well below recommended dietary allowances. Regional analysis revealed significantly different daily intake levels: highest in central Italy (233.3 ± 208.3; 95% CI: 213.2–254.1 IU/day), followed by the south (206.9 ± 178.8; 95% CI: 194.4–218.4 IU/day) and the north (189.9 ± 147.6; 95% CI: 169.8–209.8 IU/day) (*p* < 0.009). Intake decreased with age, particularly in women > 50 years of age (Figure 1): daily vitamin D intakes progressively reduced from 5*^th^* (212.5 ± 201.8 IU) to 8*^th^* decade (176.3 ± 152.1 IU). Males consistently reported a higher intake than in females across all age groups, with statistically significant differences in the 5*^th^*, 6*^th^*, and 8*^th^* decades (Figure 1). In sub-analysis in healthy subjects, daily vitamin D intake decreased from 247.8 ± 236.4 IU in the 5*^th^* decade to 202.6 ± 148.4 IU in the 8*^th^*.

We also used multivariate regression to evaluate the association of a range of clinical predictors with vitamin D intake (Table 2). This analysis revealed that male sex remained independently associated with higher daily intake (β = +44.1 IU/day, 95% CI 20.3–67.9, *p* < 0.001), while residence in Northern Italy (β = −53.0, 95% CI −82.7 to −23.2, *p* < 0.001), lower education (β = −39.2, 95% CI −72.5 to −5.9, *p* = 0.021), vegetarian diet (β = −58.4, 95% CI −103.9 to −12.9, *p* = 0.012), and vegan diet (β = −148.7, 95% CI −205.3 to −92.1, *p* < 0.001) were associated with significantly lower intake. Age was not an independent predictor after adjustment.

**TABLE 2:** Multivariable linear regression of variables associated with daily vitamin D intake.

Predictor variable	β (IU/day)	95% CI	*P*-value
**Sex**			
Male (vs. female)	+44.1	20.3 to 67.9	**< 0.001**
**Age (vs. 40–50 years)**			
51–60 years	−4.2	−37.5 to 29.1	0.806
61–70 years	−6.5	−40.3 to 27.3	0.705
71–80 years	−14.4	−59.6 to 30.7	0.530
**Geographic macro-area (vs. center)**			
North	−53.0	−82.7 to −23.2	**< 0.001**
South/Islands	−23.8	−50.6 to 3.0	0.082
**Education (vs. high school)**			
Elementary	−38.8	−71.7 to −5.8	**0.021**
Middle school	−26.9	−53.5 to −0.4	**0.047**
University	−15.0	−40.2 to 10.2	0.243
**Diet (vs. omnivore)**			
Vegetarian	−58.0	−103.3 to −12.7	**0.012**
Vegan	−149.4	−203.0 to −95.8	**< 0.001**
**Chronic disease (vs. none)**	−11.1	−32.2 to 10.1	0.305

The dependent variable (outcome variable) was daily dietary vitamin D intake (IU/day). Estimates: adjusted β (95% CI) with robust standard errors. Reference categories: female sex; age 40–50 years; Center (geographic macro-area); high-school diploma; omnivore diet; no chronic disease. CI, confidence interval. *P*-values highlighted in bold text represent statistically significant (*p* ≤ 0.05) predictors associated with daily vitamin D intake.

### 3.3 Food consumption and dietary vitamin D sources

Cheese and meat were the most frequently consumed foods (95.6% and 95.4%, respectively) (Table 3). Milk was consumed daily by 45.4% of participants, whereas fish, despite being a major vitamin D source, was consumed less frequently (only once every 14 days by 46.8% of participants). Eggs, cheese, and meat were consumed at moderate frequencies (2 times/14 days for ∼30%–40% of subjects). The most common food choices included low-fat milk, whole yogurt, parmesan cheese, beef, and tuna. A total of 65 participants reported vegetarian (87.6%) or vegan (12.3%) dietary habits.

**TABLE 3:** Food-specific vitamin D intake: user frequency, consumption patterns, and 2 weeks intake estimates.

Food	Users (%)	Mean vitamin D intake in 14 days (IU ± SD)	Types of food more frequently assumed (%)	Higher percentage intake related to higher frequency of intake in 14 days
Milk	62.7%	182.7 ± 335	Low-fat 49%; Whole 29.2%.	45.4% - 14 times
Cream	17.7%	24.8 ± 25.9	For cooking 61.3%; For dessert 38.7%.	49.8% - 1 time
Yogurt	58.5%	71.6 ± 143.9	Whole 35.7%; Low-fat 18.9%.	24.4% - 4 times
Cheese	95.6%	153 ± 111.4	Parmesan 24.0%; Mozzarella whole 15.8%.	28.9% - 2 times
Meat	95.4%	77.7 ± 76.5	Beef 39.3%; Chicken 31.1%.	41.9% - 2 times
Fish	88.8%	2279.7 ± 2143.5	Tuna 17.9%; Bluefish 16.5%.	46.8% - 1 time
Egg	89.9%	2.9 ± 2.2	Boiled 31.9%; Fried 24.9%.	41.6% - 2 times
Foods prepared with egg	80.3%	265.7 ± 280	Pasta 20.8%; Frittata 25.2%.	51.9% - 1 time
Cold cuts	82.1%	86.9 ± 109.2	Raw ham 30.8%; Baked ham 20.8%.	40.8% - 1 time
Dessert with egg/milk/yogurt	82.9% 4 cookies	303.5 ± 439.8	Ice cream 25.5%; Cookies 34%.	25% - 2 times 28.4% - 4 times
Mushrooms	43.33%	40.6 ± 91.1	Chanterelles 19.3%; Others 84.7%.	54.7% 1 time

### 3.4 Prevalence of chronic diseases and vitamin D intake

Overall, no significant difference was observed among patients with chronic diseases, osteoporosis and cardiovascular diseases were more common in older women (7*^th^*–8*^th^* decades), while cardiovascular diseases predominated among men in the 5*^th^*–7*^th^* decades (Table 4). Oncologic conditions were more frequently reported in middle-aged individuals.

**TABLE 4:** Proportion of patients with different pathological conditions stratified by gender and age.

Total population		Females (%)				Males (%)			
	*N* (%)	40–50	51–60	61–70	71–80 yrs	40–50	51–60	61–70	71–80 yrs
Osteoporosis	399 (42.3)	10.9	48.6	53.7	62.1	5.3	18.2	15.9	14.8
Cardiovascular diseases	396 (42.0)	12.5	30.5	38.8	50.8	36.8	56.6	52.4	63.0
Gastrointestinal diseases	136 (14.4)	14.1	11.3	17.8	12.3	15.8	15.9	18.3	9.9
Respiratory diseases	81 (8.6)	9.4	4.5	7.1	10.3	–	13.6	7.3	18.5
Oncologic diseases	210 (22.3)	37.5	27.1	18.1	17.9	10.5	34.1	22.0	21.0
Neurological diseases	61 (6.5)	7.8	2.3	5.9	9.2	15.8	2.3	8.5	11.1
Endocrine diseases	247 (26.2)	28.1	27.1	26.7	23.6	26.3	25.0	32.9	21.0
Renal diseases	31 (3.3)	1.6	1.7	3.2	6.2	–	4.5	3.7	1.2

**FIGURE 2:**
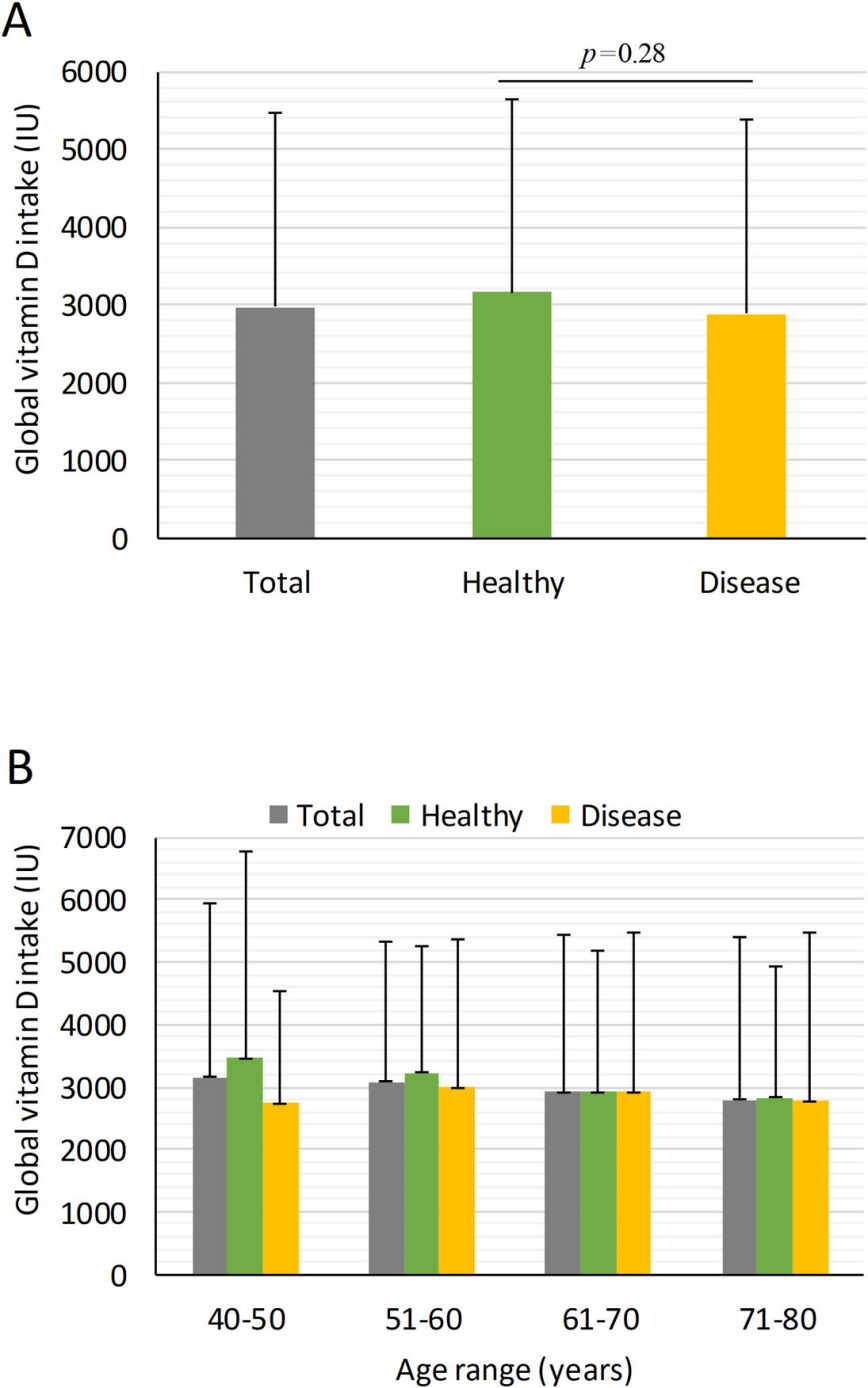
Mean global vitamin D intake in patients with pathological disorders stratified by age and gender. Global mean vitamin D intake across age ranges in patients affected by chronic pathological disorders. **(A)** Mean global vitamin D intake over 14 days in all individuals, and healthy and individuals with chronic disease; **(B)** Global mean vitamin D intake in over 14 days stratified by age decades and healthy/disease subgroups. Data are presented as mean ± SD.

**FIGURE 3:**
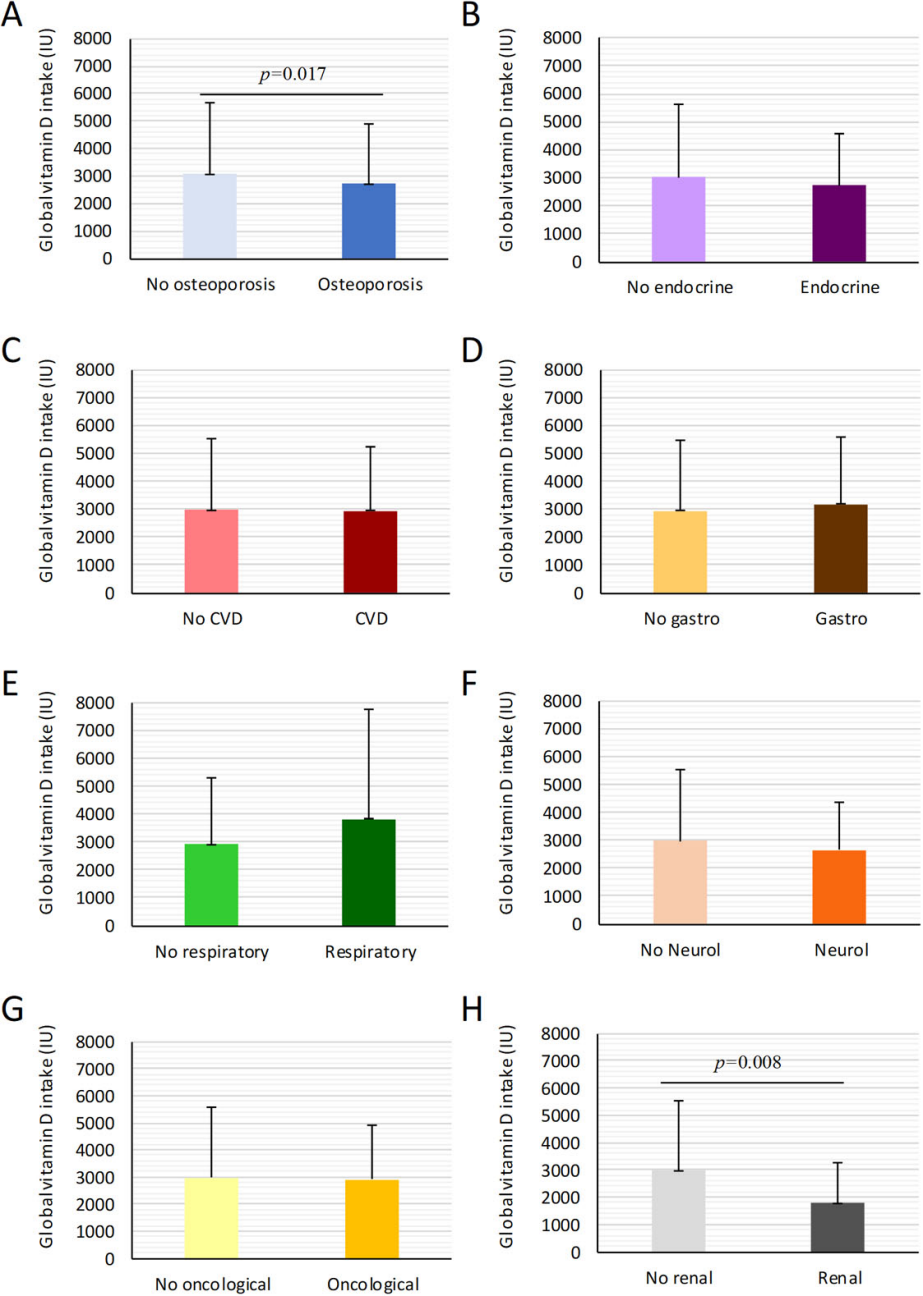
Mean global vitamin D intake in patients with and without specific pathological conditions. Comparison of mean global vitamin D intake among patients with various pathological conditions. **(A–H)** Represent different diseases including osteoporosis, renal disorders, oncologic, neurological, and endocrine conditions. Significantly lower vitamin D intake is noted in osteoporotic and renal groups, with *p*-values indicating statistical differences: *p* = 0.017 and *p* = 0.008. Data are presented as mean ± SD.

Among patients with pathological disorders, vitamin D intake was lower (2879 ± 2510 IU) than in healthy individuals (3151 ± 2481 IU), although the difference was not statistically significant (Figure 2). However, patients with osteoporosis (2543.1 ± 1904.4; 95% CI: 2293.9–2793.2 IU), and renal disorders (2210.7 ± 1711.6; 95% CI: 1929.2–2492.2 IU) had significantly lower intake than healthy individuals (*p* = 0.017 and *p* < 0.008, respectively) (Figure 3). In women, osteoporotic patients had a significant age-related decline in intake (*p* < 0.01) (Table 5). Overall, daily intake in most patient groups averaged around 200 IU/day or less, confirming critically low consumption levels.

**TABLE 5:** Mean global vitamin D intake stratified by gender age and pathological disorders.

Age range (years)	Osteoporosis	Cardio	Gastro	Respiratory	Neurological	Oncological	Renal	Endocrine	Healthy
**Female global vitamin D intake (mean ± SD)**									
40–50	2925 ± 2030	2537.9 ± 992	2896.3 ± 1102	2130.3 ± 1618	3567.4 ± 2336	3006.1 ± 1471	3560.2	2219.7 ± 1297	3238.4 ± 3567
51–60	3123.8 ± 2385	2979.7 ± 2164	2963.5 ± 2777	2952.3 ± 2038	2432.5 ± 1592	3038.1 ± 2572	2365.5 ± 1049	2963.9 ± 1919	2811.1 ± 1740
61–70	2776.4 ± 2255	3077.2 ± 2258	3298.9 ± 2482	4230.3 ± 3371	2908.6 ± 1946	2578.9 ± 1455	1953.3 ± 1421	2783.6 ± 2046	2558.8 ± 1960
71–80	2202.5 ± 1581	2411.3 ± 2074	2762.9 ± 2356	2840.8 ± 2718	2231.1 ± 1374	2857.6 ± 2334	1665.8 ± 1860	2061.7 ± 1402	2625.1 ± 2177
**Male global vitamin D intake (mean ± SD)**									
40–50	1392.9	4210.1 ± 3003	4513.5 ± 4263	–	2190.5 ± 675	–	–	2446.4 ± 1627	3840.1 ± 2837
51–60	2460.3 ± 2104	3192.3 ± 2510	3819.4 ± 2829	2899.2 ± 2245	865.5	2769.1 ± 1532	1071.8 ± 418	3777.5 ± 2595	4238.2 ± 2319
61–70	3235.8 ± 2773	3353.3 ± 3524	3016.5 ± 2205	5145.5 ± 3129	2788.8 ± 1977	3409.5 ± 1891	1571.6 ± 530	2851.1 ± 1621	3603.1 ± 2655
71–80	3689.9 ± 3919	3160.5 ± 1768	3888.1 ± 2057	5500.7 ± 5523	2927.1 ± 1745	3123.5 ± 2406	1223.7	3172.1 ± 1841	3143.9 ± 1922

Logistic regression analysis revealed that male sex was protective against very low intake (< 200 IU/day) (OR 0.72, 95% CI 0.54–0.96, *p* = 0.025) (Table 6). Conversely, Northern Italian residence (OR 1.61, 95% CI 1.20–2.16, *p* = 0.002), lower education (OR 1.45, 95% CI 1.06–1.97, *p* = 0.021), vegetarian diet (OR 1.86, 95% CI 1.14–3.04, *p* = 0.012), and vegan diet (OR 3.89, 95% CI 2.12–7.15, *p* < 0.001) were associated with higher odds of very low intake. Chronic disease status was not an independent predictor.

**TABLE 6:** Multivariable logistic regression analysis of predictor variables associated with very low (< 200 IU/day) vitamin D daily intake.

Predictor	Category	OR	95% CI	*P*-value
Sex	Male vs. female	0.72	0.54–0.96	**0.025**
Region	North vs. center	1.61	1.20–2.16	**0.002**
Education	Elementary vs. high/univ	1.45	1.06–1.97	**0.021**
	Middle vs. high/univ	1.12	0.82–1.52	0.478
Diet	Vegetarian vs. omnivore	1.86	1.14–3.04	**0.012**
	Vegan vs. omnivore	3.89	2.12–7.15	**< 0.001**
Disease	Chronic vs. healthy	1.08	0.79–1.47	0.612
Age	51–80 vs. 40–50 years	1.05	0.78–1.41	0.742

Outcome: odds of very low vitamin D intake (< 200 IU/day). Adjusted for sex, age, region, education, diet, and disease status. Data presented as odds ratios (OR) and with 95% confidence intervals (CI) and relative *p*-values. *P*-values highlighted in bold text represent statistically significant (*p* ≤ 0.05) variables associated with very low vitamin D daily intake.

### 3.5 Influence of socioeconomic factors

Vitamin D intake varied by education and occupation. Individuals with university or high school diplomas had significantly higher intake levels than those with only primary or middle school education (*p* < 0.002). Professionals, freelancers, and managers reported higher intake levels than artisans, farmers, and housewives (*p* < 0.03). Vegetarians and vegans had significantly lower daily vitamin D intake (152.1 ± 162.9 IU; 95% CI: 73.1–188.9 and 83.6 ± 57.4; 95% CI: 43.8–123.3 IU/day, respectively) compared to the general population (*p* < 0.004).

## 4 Discussion

Results from the present survey confirm and extend our previous findings demonstrating that in an adult Italian population (females or males), that there is a lack of vitamin D intake. This condition was demonstrated either in healthy subjects or in patients with different pathological disorders. The reduction in vitamin D intake was widely observed across different geographical areas of Italy: nevertheless, with respect to macro area of Center, North and South areas were characterized by a more marked lower intake of vitamin D. Our data therefore show that geographical area emerged as an independent predictor of vitamin D intake in Italy, consistent with earlier studies reporting regional dietary differences (13). Other interesting outcomes observed from the present survey were that socioeconomic conditions and education level may shape vitamin D intake: low levels were seen in artisans, farmers, housewives, workers, and secondary/middle school or primary/elementary school diploma, respectively. These results highlight the importance of education and socioeconomic status as key predictors of low vitamin D intake in the Italian population. The knowledge and awareness of a correct nutrition may influence in our opinion the choice of foods and consequently the vitamin D intake. Previous data (13) regarding the markedly low vitamin D intake in subjects following vegetarian or vegan eating habits is again confirmed by our new data. Taken together, our analyses indicate that diet pattern, education, and region of residence are the main predictors of inadequate intake in Italy.

These findings carry practical implications: our results support the potential role of food fortification and/or supplementation strategies in the Italian context, where mean intake remains well below recommendations.

Analyses adjusting for multiple factors showed that male sex was independently associated with higher vitamin D intake, while residence in Northern Italy, lower education levels, and vegetarian or vegan diets were linked to significantly lower intake. Logistic regression further indicated that men had reduced odds of very low intake (< 200 IU/day), whereas Northern residence, low education, and vegetarian or vegan diets substantially increased this risk.

The reduced assumption of vitamin D was particularly noticeable (daily vitamin D intake ≤ 200 IU) in patients with osteoporosis, and renal, neurological, endocrine and oncologic disorders. The sample size of groups was adequate (with the only exception of patients with renal disease) highlighting the role that a reduced vitamin D intake may play in the development of hypovitaminosis D even in pathological conditions. With advancing age, the global assumption of vitamin D in patients with pathological disorders was seen to progressively decrease, with the exception of respiratory and endocrine diseases. In the female population a similar trend was observed in osteoporotic patients, and in neurological and renal disorders: in the 4^th^ decade of age the daily vitamin D intake in osteoporotic females, and in women with renal and neurological disorders were 157.3, 119, and 159.4 IU, respectively. The different behavior of vitamin D intakes in males may be explained by the smaller number of patients included in each age range. Together, these findings underline consistent, independent sociodemographic and dietary predictors of inadequate vitamin D intake in Italy, and highlight high-risk groups where preventive strategies may be most urgently needed.

The relationship between hypovitaminosis D and bone disorders is fairly well-established (37, 38). The clinical consequences of severe deficiency of vitamin D are rickets in children and osteomalacia in adults (39–41). In addition to nutritional osteomalacia, hypovitaminosis is also involved in the development of reduced intestinal calcium absorption and secondary hyperparathyroidism (42). Together, with poor sun exposure, inadequate food intake markedly contributes to hypovitaminosis D (16).

In post-menopausal women, the consequent increased bone resorption results in a decrease in bone mass and increased risk of bone fracture (43). Several studies and systematic reviews have found insufficient or conflicting evidence to support the beneficial effect of the use of supplemental vitamin D to induce musculoskeletal health in adults living in the community (44–48). Factors linked to vitamin D effects may be related to dosage regimens, frequency, formulation, duration of treatment, and patients’ characteristics and demographics, including serum baseline, lifestyle habits, ethnicity, and genetics (49). The ineffectiveness of high-dose, supplemental vitamin D on BMD may be explained by baseline 25(OH)D levels that may already be sufficient to support normal bone health (50). These findings do not apply to subjects with extremely low vitamin D levels or osteoporosis: considering seasons/sun-light exposure and dietary vitamin D intakes, in adults > 50 years old and those with a higher bone fracture risk, daily intake of 800–1,000 IU of vitamin D, including supplements if necessary, are recommended (51).

The low number of patients with renal disease does not allow detailed considerations, also because the severity of chronic kidney disease (CKD) is not specified. In any case, many causes may promote in CKD vitamin D deficiency: age, female sex, adiposity, low physical activity, proteinuria, impaired 25(OH)D tubular reabsorption, reduced skin synthesis and intake of vitamin D (52). Together, with decreased kidney function, a decrease in 1,25(OH)2D leads to hypocalcemia and secondary hyperparathyroidism, which are the main causes of secondary osteoporosis. Consequently, checking and supplementing low serum 25(OH)D levels in CKD patients is recommended (53). Considering our data showing low levels of vitamin D intake, a specific care to improve the assumption of vitamin D rich foods would also be considered.

Emerging evidence from observational epidemiological studies of significant inverse associations between vitamin D status and a range of non-skeletal diseases such as cardiovascular disease (25–27, 54), cancer (30). In patients of present survey who referred cardiovascular disease (high blood pressure included), the mean global daily vitamin D intake was 211 IU, significantly lower with respect to recommended dose: the intake was particularly reduced in female population, 199 IU. With aging, in males a decrease in vitamin D intakes was observed from 5^th^ to 8^th^ decades. The ViDA study, a randomized, double-blind, placebo-controlled trial, shows that high-dose monthly vitamin D supplementation does not reduce the incident cardiovascular events (55).

In another intervention clinical trial, the Vitamin D and Omega-3 Trial (VITAL), supplementation with vitamin D (cholecalciferol at a dose of 2,000 IU per day) was also not found to be associated with a lower risk of major cardiovascular events (56). Similar results were observed in patients with cancer: the cumulative incidence of invasive cancer of any type (breast, prostate and colorectal cancer) and death from cancer did not differ significantly between vitamin D and placebo groups (56, 57). On the other hand, beneficial effects were reported in ViDA study for some intermediate outcomes such as bone mineral density, lung function and arterial function in participants with 25(OH)D concentrations of ≤ 30 nmol/L, suggesting that vitamin D supplementation could be beneficial in people who are vitamin D deficient (58).

The discrepancy between, significant correlation between hypovitaminosis D and increased risk of cardiovascular disease and cancer, and, on the other, the absence of a significantly lower incidence of invasive cancer of any type or a composite of major cardiovascular events (myocardial infarction, stroke, and death from cardiovascular causes) is not easily explainable. Different age of recruited population, length of observational studies, variable vitamin D status, presence of comorbidities, different risk factors may be hypothesized.

Low levels of vitamin D intake was also observed in patients with endocrine disorders. Considering that this group includes also patients with type 2 diabetes, the role that vitamin D deficiency may increase the risk of type 1 and type 2 diabetes must be considered (21). To date, the relationship between vitamin D and diabetes are not fully understood (21, 59).

Observational and interventional studies support the efficacy of vitamin D for the prevention of type 1 and type 2 diabetes onset and development (60). In addition to the aforementioned disorders, other diseases are associated with poor vitamin D status (15). Observational studies suggest a link between low vitamin D levels and several inflammatory lung diseases or impaired lung function (61), increased risk of infection or risk of autoimmune diseases such as inflammatory bowel diseases (62). Our data demonstrating reduced levels of vitamin D intake in patients with respiratory, neurological and gastrointestinal disorders suggest a possible role that hypovitaminosis D may play in the development of these diseases. Strictly connected to these aspects is the relationship between hypovitaminosis D and mortality.

In an IPD (individual participant data) meta-analysis using standardized measurements of 25(OH)D, in 26,916 individuals from a European consortium, an association between low vitamin D status and increased risk of all-cause mortality was observed (63). Re-analysis of results based on older studies, including more vitamin D–deficient subjects indicated a small but significant reduction in mortality (64, 65). These data reveal that the diversified effects of vitamin D supplementation may be due also to different vitamin D status, and indirectly the importance of correct nutritional approach.

### 4.1 Study limitations

There are several weaknesses of the present study that need to be acknowledged. First, the cross-sectional design prevents any inference of causality. Second, dietary intake was assessed using a self-reported FFQ, which is subject to recall errors and social desirability bias and may lead to both underestimation and overestimation of actual intake. Third, serum 25(OH)D was not measured, so we could not evaluate the relationship between reported intake and vitamin D status. Fourth, although we adjusted for a range of covariates (sex, age, macro-area, education, occupation, diet pattern, chronic disease), residual confounding cannot be excluded (i.e., sunlight exposure, outdoor physical activity, BMI, or seasonality). Finally, disease subtype indicators were based on self-report, and some subgroups were small.

## 5 Conclusion

In conclusion, data from the present survey confirm our previous results showing an extremely low vitamin D intake in the adult Italian population. Moreover, in a wide group of patients with different pathological disorders, the assumption of vitamin D was found to be significantly lower with compared to healthy individuals. This observation was seen in both females and males: moreover, the reduced vitamin D intake tended to decrease with increased age.

This study suggests that the implementation of educational healthcare policies, starting from childhood and continuing into adulthood, to promote a correct dietary approach rich in vitamin D. This type of diet, including mainly fish, milk, and its derivatives, would integrate the nutritional model inspired by the traditional eating habits of countries bordering the Mediterranean sea, known as the “Mediterranean diet,” followed predominantly by Italian population. Considering the extremely low intake in patients with osteoporosis and other pathological disorders as cardiovascular diseases and cancer, a vitamin D-rich diet could play a significant role in preventing their development (66).

Fortification of certain foods (such as milk and cheese) with vitamin D may represent an alternative approach to non-feasibility of dietary changes (67–69). In specific circumstances, especially in at-risk groups like children, pregnant women, and the elderly, vitamin D supplementation remains a crucial strategy to minimize the risk of hypovitaminosis (70).

Dietary vitamin D intake in Italian adults remains far below recommended levels. Independent variables correlating with lower intake include Northern macro-area, lower education, and plant-based dietary patterns. Future intervention studies should monitor serum 25(OH)D, evaluate fortification and supplementation strategies in trials, and inform policy on the design and reach of national fortification programs and targeted education for at-risk groups, also introducing specific national guidelines.

## Data Availability

The original contributions presented in this study are included in this article/supplementary material, further inquiries can be directed to the corresponding author.
