# Correction: Vitamin D intake in Italian healthy subjects and patients with different pathological disorders

**DOI:** 10.3389/fnut.2025.1756282

**Published:** 2025-12-17

**Authors:** Ranuccio Nuti, Luigi Gennari, Guido Cavati, Carla Caffarelli, Bruno Frediani, Stefano Gonnelli, Concetta Laurentaci, Giulia Letizia Mauro, Nazzarena Malavolta, Giovanni Minisola, Maria Punzo, Anna Capozzi, Monica Pinto, Fabio Vescini, Edoardo Conticini, Giammarco De Mattia, Agostino Gaudio, Colin Gerard Egan, Daniela Merlotti

**Affiliations:** 1 Department of Medicine, Surgery and Neurosciences, University of Siena, Siena, Italy; 2 Azienda Sanitaria Matera, Matera, Italy; 3 Dipartimento delle Discipline Chirurgiche, Oncologiche e Stomatologiche, University of Palermo, Palermo, Italy; 4 Casa di Cura Madre Fortunata Toniolo, Bologna, Italy; 5 Ospedale San Camillo, Rome, Italy; 6 ASP Cosenza, Cosenza, Italy; 7 Policlinico Gemelli, Rome, Italy; 8 Istituto Nazionale Tumori IRCCS Fondazione G. Pascale, Napoli, Italy; 9 Department of Oncology, University of Udine, Udine, Italy; 10 Department of Medical and Surgical Specialties, University of Pisa, Pisa, Italy; 11 Department of Clinical and Experimental Medicine, University of Catania, Catania, Italy; 12 CE Medical Writing SRLS, Pisa, Italy; 13 Department of Medical Sciences, Azienda Ospedaliera Universitaria Senese, Siena, Italy

**Keywords:** vitamin D intake, hypovitaminosis D, dietary assessment, chronic disease, Italian population

Affiliation 6 in the article was erroneously given as “Istituto Sant'Anna Crotone, Crotone, Italy” as published. The correct affiliation should read as “ASP Cosenza, Cosenza, Italy.”

The original version of this article has been updated.

